# Measuring the willingness to share personal health information: a systematic review

**DOI:** 10.3389/fpubh.2023.1213615

**Published:** 2023-07-20

**Authors:** Marcello Benevento, Gabriele Mandarelli, Francesco Carravetta, Davide Ferorelli, Cristina Caterino, Simona Nicolì, Antonella Massari, Biagio Solarino

**Affiliations:** ^1^Department of Interdisciplinary Medicine, University of Bari, Bari, Italy; ^2^Department of Economics, Management and Business Law, University of Bari, Bari, Italy

**Keywords:** health data, personal health information, secondary data uses, privacy, data sharing

## Abstract

**Background:**

In the age of digitalization and big data, personal health information is a key resource for health care and clinical research. This study aimed to analyze the determinants and describe the measurement of the willingness to disclose personal health information.

**Methods:**

The study conducted a systematic review of articles assessing willingness to share personal health information as a primary or secondary outcome. The review followed the Preferred Reporting Items for Systematic Reviews and Meta-Analysis protocol. English and Italian peer-reviewed research articles were included with no restrictions for publication years. Findings were narratively synthesized.

**Results:**

The search strategy found 1,087 papers, 89 of which passed the screening for title and abstract and the full-text assessment.

**Conclusion:**

No validated measurement tool has been developed for willingness to share personal health information. The reviewed papers measured it through surveys, interviews, and questionnaires, which were mutually incomparable. The secondary use of data was the most important determinant of willingness to share, whereas clinical and socioeconomic variables had a slight effect. The main concern discouraging data sharing was privacy, although good data anonymization and the high perceived benefits of sharing may overcome this issue.

## 1. Introduction

The spread of information technologies has challenged assumptions about privacy and confidentiality, leading to feelings of discomfort, threat, and mistrust (1). This highlights the importance of personal data, which are defined as “any information relating to an identified or identifiable natural person,” according to Article 4 of the General Data Protection Regulation, a Regulation in EU law on data protection and privacy in the EU and the European Economic Area.

Studies have also highlighted the divergence between attitudes and behavior related to privacy, known as the “privacy paradox” (2–5). Evidence indicates that individuals are willing to share their personal information for relatively small rewards, which contrasts with the privacy concerns showed in polls and surveys (6). The paradox has been especially studied in the context of e-commerce and social media, as the understanding of privacy attitudes and behaviors has significant implications for the huge collectors of personal information (7).

Since the past decade, many countries have introduced digital health transformation (8, 9). This process proceeded with the progressive adoption of new technologies; development of telemedicine; and diffusion of wearable electronic devices, consumer-oriented apps, and services (10–14). A direct consequence of health digitalization is the availability of a variety of data, many of which can be considered personal data concerning health or personal health information (PHI) (15). Such complex and rich data may drive the development of high-performing big data and artificial intelligence (AI) systems in health care, which are also fundamental in producing new insights for clinical research (16, 17). For this reason, digital health and health-data management have become a priority, firmly embedded in EU policy and funding goals (18). However, even if PHI represents an inalienable resource, personal preference in disclosing such information may limit access to PHI (8, 19–25). During the coronavirus disease 2019 (COVID-19) pandemic, the usefulness of sharing PHI was observed: PHI has been fundamental for contact tracing, managing the vaccination campaign, and other public health interventions (26, 27). At the same time, many observers registered several risks to privacy triggered by the pandemic (28, 29).

Given the importance of PHI in the age of data-driven health care policy and clinical research, health sciences should elucidate the theoretical explanations of privacy attitudes and behaviors (30), which can help ensure ethical patient-centered policies and influence the development of health care practice. One of the key stages of this challenge in the health care context is the investigation of the personal willingness to share PHI. To the best of our knowledge, there are no validated tools to measure the willingness to share PHI. Thus, we conducted this systematic review to identify the tools that have been used to measure the willingness to share PHI and to investigate the clinical, demographic, and neuropsychological factors associated with such willingness.

## 2. Methods

This systematic review was conducted by following the Preferred Reporting Items for Systematic Review and Meta-Analyses (PRISMA) guidelines (31).

### 2.1. Search strategy

We used a systematic search strategy to conduct a two-step literature search. The first step, started on February 16, 2022, involved searching the Medline/PubMed, Web of Science, and Scopus databases using the following strings: (shar* OR disclos*) AND (personal OR electronic) AND (“health data” OR “health record” OR “health information”) AND (willing* OR desir* OR eager* OR disposit* OR inclinat*).

As a second step, two of the investigators included studies searched through the reference lists of included/excluded papers or relevant reviews with no limitation on the year of publication. The identified papers were then screened based on their titles and abstracts, and papers that passed the screening were assessed for eligibility by full-text reading. Discrepancies were resolved by consulting a third opinion or conducting Delphi rounds with all the authors when necessary. The review protocol was registered with PROSPERO (registration number: CRD42022341477).

### 2.2. Inclusion and exclusion criteria

Papers were included if the studies measured the willingness to share PHI. We excluded the articles written in languages other than English and Italian, reviews, case reports, and those whose full text could not be obtained even after contacting the corresponding author.

### 2.3. Data extraction

Titles and abstracts were independently screened by two reviewers in duplicate to determine whether the retrieved studies met the inclusion criteria outlined above. The full papers were obtained for studies that appeared to have met the inclusion criteria, or if screening of the title and abstract resulted in uncertainty. The full texts of all papers that passed the screening were independently assessed for eligibility by two reviewers. Discrepancies were resolved with a third reviewer or Delphi rounds, if required. We used a standardized form to extract data from the included studies to assist in study quality and evidence synthesis. Extracted information included the following: focus of the study, participant characteristics, measurement of willingness to share PHI, criteria used to validate the final judgment, and authors’ conclusions, as well as information required for the assessment of the risk of bias. Data extraction was performed by two reviewers independently and in duplicate. A third reviewer was consulted, if necessary.

### 2.4. Data charting

The aims and main results of the selected studies were briefly described. The study design was as follows: when the authors used a questionnaire with no open-ended questions, interviews were conducted using an open-ended questionnaire.

### 2.5. Quality assessment

Two independent reviewers performed a quality assessment for the systematic reviews of qualitative evidence to assess the quality of the selected studies. The quality assessment tool was drawn directly from Appendix D of Hawker et al. (32) The tool consisted of nine questions, each of which can be answered with “good,” “fair,” “poor,” or “very poor,” which we subsequently converted into a numerical score (from 1 to 4 points). Hence, every study received a score (from 9 to 36 points) that defined their overall quality grade: high quality, 30–36 points; medium quality, 24–29 points; and low quality, 9–24 points. The nine questions in the tool were as follows:

1. Abstract and title. Did they provide a clear description of the study? Good: structured abstract with full information and clear title. Fair: informative abstract. Poor: inadequate abstract. Very poor: no abstract.
2. Introduction and aims. Was there a good background section and a clear statement on the aims of the research? Good: full but concise background to discussion/study containing up-to-date literature review and highlighting gaps in knowledge; clear statement of aim and objectives, including research questions. Fair: background, literature review, and research questions outlined. Poor: some background but no aim/objectives/questions or aims/objectives but an inadequate background. Very poor: no mention of aims/objectives; no background or literature review.
3. Methods and data. Is the method appropriate and clearly explained? Good: method is appropriate and described clearly (e.g., questionnaires included); clear details of data collection and recording. Fair: method is appropriate; the description could be better. Poor: questionable whether the method is appropriate; the method is described inadequately, with little description of the data. Very poor: no mention of method and/or method inappropriate, and/or no details of data.
4. Sampling. Was the sampling strategy appropriate to address the aims? Good: details (age/sex/race/context) of who was studied, how they were recruited, and why this group was targeted; the sample size was justified for the study; response rates were shown and explained. Fair: sample size justified; most information is given but some are missing. Poor: sampling mentioned but with few descriptive details. Very poor: no sample details.
5. Data analysis. Was the description of the data analysis sufficiently rigorous? Good: clear description of how the analysis was carried out; description of how themes were derived and of respondent validation or triangulation. Fair: descriptive analysis. Poor: minimal details of the analysis. Very poor: no discussion of the analysis.
6. Ethics and bias. Were ethical issues addressed and was the necessary ethical approval gained? Was the relationship between researchers and participants adequately considered? Good: ethics: when necessary, issues of confidentiality, sensitivity, and consent were addressed; bias: the researcher was reflexive and/or aware of their own bias. Fair: lip service was paid to the above (i.e., these issues were acknowledged). Poor: brief mention of issues. Very poor: no mention of issues.
7. Results. Is there a clear statement about the findings? Good: findings are explicit, easy to understand, and in a logical progression; tables, if present, are explained in text; results relate directly to aims; sufficient data are presented to support the findings. Fair: findings mentioned but more explanation could be given; data presented relate directly to results. Poor: findings presented haphazardly, not explained, and do not progress logically from the results. Very poor: findings not mentioned or unrelated to aims.
8. Transferability or generalizability. Are the findings transferable (generalizable) to a wider population? Good: context and setting of the study are described sufficiently to allow comparison with other contexts and settings, plus a high score in sampling (Q4). Fair: some context and setting are described, but more is needed to replicate or compare the study with others. Poor: minimal description of contexts/settings. Very poor: no description of context/setting.
9. Implications and usefulness. How important are the findings to policy and practice? Good: contributes something new and/or different in terms of understanding/insight or perspective; suggests ideas for further research and implications for policy and/or practice. Fair: two of the above. Poor: only one of these. Very poor: none of the above.

## 3. Results

### 3.1. Search, screening, full text assessment

**Figure 1:**
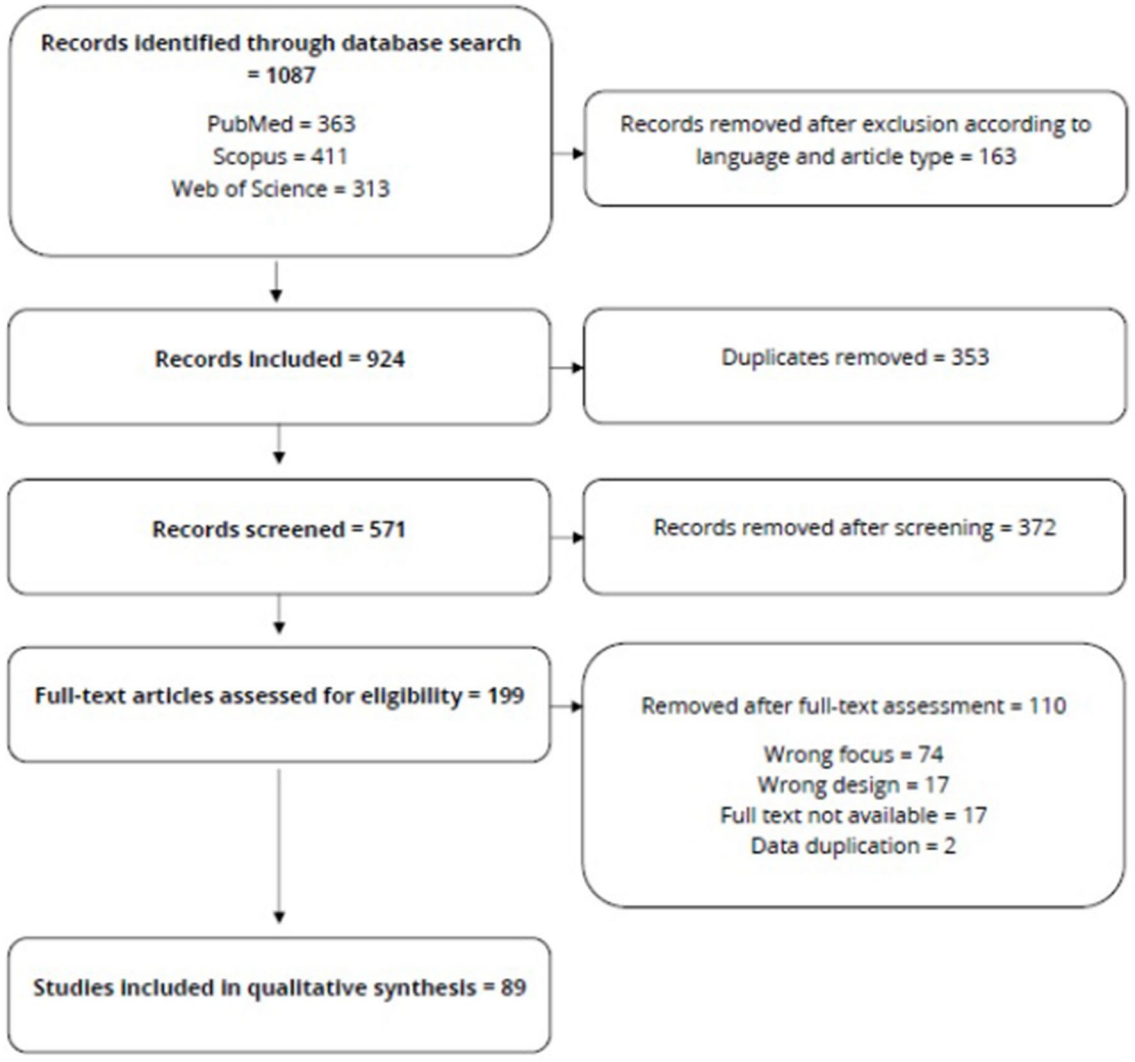
Flow diagram of papers selection.

Our database search identified 1,087 studies. After applying the inclusion criteria (language and type of paper), the authors included 924 papers, 353 of which were duplicates. The authors screened 571 studies based on the titles and abstracts; we identified 199 papers eligible for full-text assessment. The 89 papers that passed the assessment were included in the data extraction Figure 1 depicts the flow of the paper selection process.

### 3.2. Quality assessment

The quality assessment is available in the Supplementary material. The mean overall quality score was 25.4/36 (range 13–36), corresponding to the medium quality rate. The lowest average scores were for the sampling (average of 2.2/4) and ethics and bias items (average of 2.2/4). The highest average scores were for the introduction/aims (average of 3.3/4) and data analysis items (average of 3.2/4).

### 3.3. Methods analysis

The charted data are available in the Supplementary material. Among the 89 selected papers, 66 presented the results of a survey, 22, interviews, and one, an analysis of patients’ preferences in informed consent acquisition. Nine papers presented hypothetical scenarios to the participants, and 13 invited them to join focus groups. The recruiters were authors in 51 cases, a market research company in 25 cases, and a public/non-profit organization in 13 cases. The recruiters used the in-person approach in 21 cases, email announcements/social media posts/posted fliers in 19 cases, and telephone calls or letters in six cases. Forty-six papers used qualitative content analysis, factorial analysis, structural equation models, cluster analysis, or inductive approach; 43 papers used only descriptive and inferential statistics. The studies required online participation in 47 cases, in-person sessions in 28 cases, telephone sessions in three cases, and mixed methods in the other cases.

## 4. Discussion

As almost all (88/89) of the selected papers investigated willingness to share PHI through surveys or interviews, it was not possible to summarize the results with a meta-analysis. About half of the selected papers employed qualitative analysis (46/89), and the rest (43/89) used only descriptive or inferential statistics. The overall quality of the studies ranged from high (36/36) to low (13/36); however, the average overall quality grades corresponded to medium (25.4/36).

Of the 89 selected papers published between 2006 and 2022, only two were published before 2009. This finding suggests that interest in PHI management increased along with the digitalization of health care and the more general diffusion of information technologies, which emerged in the past two decades (30). The years 2019, 2020, and 2022 registered high publication rates (13, 18, and 13, respectively), and more than half of the studies (56/89) were published between 2019 and 2022. As the search was completed in June 2022, we assumed that the publication rate in 2022 is largely underestimated.

The COVID-19 pandemic has aroused debate concerning PHI. Public health authorities recognized the need to carry out contact-tracing and personal restrictions, which entailed collecting and sharing personal data and PHI (e.g., personal localization, results of SARS-CoV-2 tests) through and between public organizations (17, 33, 34). Consequently, the management of PHI during the pandemic raised serious privacy concerns (35, 36). However, patients have been more comfortable sharing PHI during the COVID-19 pandemic, especially with care institutions and researchers (37). This may be linked to the burden of the pandemic, which redefined people’s perception of benefits.

Indeed, a vast amount of PHI has been necessary to cope with the pandemic while managing contact tracing and vaccination efforts. In the same period, researchers have also hypothesized the use of crowdsensing for research, which consists of collecting people’s location information *via* mobile sensing devices (38). Although this hypothesis seemed to threaten the right to personal privacy, many other forms of crowdsensing (e.g., real location information sent by mobile phones) had been used, even including for commercial purposes (39). The pandemic has also triggered efforts to increase health literacy, which positively influences the willingness to share PHI (40). Owing to the spread of COVID-19, many health providers adopted telemedicine, which allowed the delivery of health care services without any close contact (41, 42). Whether and how telemedicine may have provided a vast amount of digital PHI and influenced the likelihood of sharing PHI remain unclear and deserve a deeper analysis.

In general, increasingly digitalized health enables new far-reaching opportunities for the secondary use of PHI. Secondary uses may be categorized as for public health (e.g., public health surveillance), research, health care quality improvement [e.g., adoption of electronic health records (EHR)], and commercial (e.g., marketing, health insurance) purposes (43). Our results clearly showed that the secondary use of PHI represented one of the most important determinants of the willingness to share PHI.

People tend to be willing to share their PHI for research (44–52). However, previous results may reflect a sort of selection bias: responders who declare a good willingness to share PHI for research purposes are more likely to already be actively participating in research. In other words, we could not determine how willingness to participate in research could influence the willingness to share PHI for research, even if they seem to be mutually related. The only way to reduce the impact of this bias would be to increase sample representativeness. Given this limitation, the willingness to share PHI for research has been associated with sociodemographic features (white race, higher educational attainment, lower religiosity, health literacy), but also with the perception of more research benefits (15, 53–55). Biobanks and other large research projects have registered similar results (53, 56).

However, the type of disclosed information still determines the willingness to share for research. For example, a sample of 36,268 individuals from 22 countries showed a low willingness to share DNA data for research (57). This result seems to confirm the theory of “genetic exceptionalism,” according to which genetic data should be treated separately from other medical information (58). Meanwhile, in the same study, responders experienced with genetics and those who self-defined as “genetic exceptionalist” were much more willing than others to disclose genetic data for research (57). Moreover, a recent study demonstrated that disclosing genetic data is acceptable if anonymity is ensured (59).

Regarding health care quality improvement, an increasing number of countries are adopting EHR, a tool that is expected to enable safe and high-performing data exchange among health policy-makers, providers, and patients (60). Patients are willing to share their PHI using EHR, especially with providers involved in their care, and to facilitate health information exchange between them (61–68). Nevertheless, selecting the PHI to share with EHR makes patients more comfortable and increases their willingness to adopt EHR (69). Among older adults, EHR acceptance is positively influenced by a higher degree of multimorbidity, higher number of prescribed medications, higher number of hospital admissions, and living with a chronic illness, whereas a pessimistic attitude and lack of joy in life, as indicators of depressive mood, have a negative impact (70).

Meanwhile, commercial use is a strong determinant of unwillingness to share PHI (71–74). Nonetheless, researchers and health care organizations often need commercial partners to recruit samples and obtain suitable cloud servers or other technological infrastructures, especially while building big data (75). Users of health and fitness apps are also aware that these apps transmit user data to several third parties (76). For example, among the reviewed papers, the sample was recruited by a private research company in 25/89 cases. One major concern regarding third-party commercial use of PHI is the risk of discrimination (insurance and employment), especially related to the sharing of genomic data and mobile/wearable device data (59, 66). Insurance discrimination may act subtly; some United States insurance companies have started to move to interactive life insurance models by providing discounts to customers who share fitness data *via* monitoring devices (77). In this context, the fear of possible genetic insurance discrimination looms large in the public imagination, and empirical data suggest that this holds people back from undergoing testing (77).

Another issue relates to employees’ privacy being challenged in several ways. For example, during the pandemic, employers faced many burning questions on data protection, including the conditions under which they could process employees’ PHI to ensure health safety in the workplace (78). The risk of discrimination may be even more concrete while building AI into PHI databases, as AI predictive models can contain several layers of potential bias (79).

Another secondary use of PHI is peer-to-peer information exchange, which mainly regards patients with chronic or serious diseases. Independent of the secondary users of PHI, our findings demonstrated that the type of disease significantly influenced individuals’ perceptions of usefulness, accessibility, psychological risk, privacy concerns, stigma, and willingness to share PHI (80–82). For example, people living with HIV and adolescents with diabetes typically participate in peer-to-peer digital groups to improve their daily self-management (83, 84). Despite this, people living with HIV accept data sharing more willingly depending on the efforts expended to ensure the confidentiality of HIV-related data, as the stigma surrounding HIV prompts hesitancy in sharing PHI (85).

Many authors stated that both the general population and patients are more willing to share their non-psychiatric medical information than psychiatric information (13, 48, 52, 86, 87). Meanwhile, people with mental illness have shown more willingness to share PHI even when it entails disclosing their mental disease (50). Hence, even if the stigma surrounding psychiatric conditions may inhibit the willingness to share medical information, the benefits of sharing perceived by psychiatric patients can overcome their sense of uncertainty (88).

Patients with cancer are also more willing to share their inherited genetic information and other medical details than daily life or identity information (69, 89). The information-sharing preferences of patients with cancer or cancer survivors are driven mainly by the purpose of information reuse and type of data shared (90, 91). The likelihood of sharing PHI among cancer survivors may rely on the altruistic belief that the data could benefit others (91). A similar “altruism” might have also promoted participation in public health efforts during the COVID-19 pandemic (33). However, the influence of altruism on sharing PHI may also depend on stressful experiences, such as surviving cancer or living through the pandemic.

The influence of low socioeconomic status on willingness to share PHI showed some contradictory results. Green et al. observed that a low socioeconomic status may negatively influence the willingness to share PHI (92). Otherwise, several authors have indicated that low-income people largely support the sharing of PHI if doing so demonstrates benefits (93–96). The contradictory influence of low socioeconomic status may be indirectly due to the observed positive influence of health literacy and trust in health organizations, which in turn may be linked to socioeconomic status (34, 70).

The major concerns regarding PHI sharing are data misuse and data breach (48, 51, 53, 56, 69, 97). As such, strong underlying privacy views affect willingness to share PHI, and anonymization assumes a crucial role (98–107). Hence, trust in data security and awareness of data-storing systems can enhance participation in data sharing and research (108–114).

Finally, the observed reticence about sharing non-medical information (e.g., socioeconomic status) highlights the importance of shared data (12, 115). An increasing number of researchers and research institutions consider AI as an opportunity for future development in many human activities. However, it requires immense volumes of data (big data), represented by PHI in the health care context.

We found that although the willingness to share PHI seemed to meet the interest of the research community, the methods used to measure it varied, making outcomes mutually incomparable. This represents a significant limitation of studying such an issue. Given the importance of health data sharing, a standardized method to assess the willingness to share PHI and its determinants is necessary.

## 5. Conclusion

Despite the progressive digitalization of health care and the crucial role of health data in health care and research, until date, no validated measurement tool has been developed for willingness to share PHI. The reviewed papers measured such willingness through surveys, interviews, and questionnaires, which were mutually incomparable. The secondary use of PHI was the major determinant of the likelihood of sharing PHI, whereas clinical and socioeconomic variables had slight effects. Privacy was the main concern discouraging data sharing. However, good data anonymization and the high perceived benefits of sharing may overcome such concern. A better understanding of the phenomenon may drive the development of patient-centered digital health care and more ethical and effective clinical research.

## Author contributions

MB and GM conceptualized the research goals and wrote the original draft. FC, CC, and SN conducted the investigation. DF, AM, and BS reviewed and edited the paper. AM and BS coordinated the research activity. All authors contributed to the article and approved the submitted version.

## Funding

This work was supported by the Horizon Europe Seeds program (ID S16—CUP H91I21001610006).

## Conflict of interest

The authors declare that the research was conducted in the absence of any commercial or financial relationships that could be construed as a potential conflict of interest.

## Publisher’s note

All claims expressed in this article are solely those of the authors and do not necessarily represent those of their affiliated organizations, or those of the publisher, the editors and the reviewers. Any product that may be evaluated in this article, or claim that may be made by its manufacturer, is not guaranteed or endorsed by the publisher.
