# Supplementary material for: Measuring the willingness to share personal health information: a systematic review
Source: Front Public Health. 2023 Jul 20;11:1213615. doi: 10.3389/fpubh.2023.1213615 (PMC10397406; doi:10.3389/fpubh.2023.1213615)
Supplement: Supplementary file 1 [file Table_1.docx]

Supplementary Material

# Supplementary Tables

## Supplementary Table 1

| **Author, Year, Country** | **Abstract/title** | **Intro/aims** | **Method and data** | **Sampling** | **Dana analysis** | **Ethics and bias** | **Results** | **Transferability** | **Implications** | **Total** |
| --- | --- | --- | --- | --- | --- | --- | --- | --- | --- | --- |
| Lee CMY *et al.,* 2022, Australia | 4 | 4 | 3 | 2 | 3 | 2 | 2 | 4 | 1 | 25 |
| Tosoni S *et al,* 2022, Canada | 4 | 3 | 4 | 1 | 4 | 2 | 2 | 4 | 1 | 25 |
| Cherif E *et al,* 2022, France | 2 | 3 | 3 | 2 | 4 | 2 | 2 | 4 | 2 | 24 |
| Belfrage S *et al*, 2022, Sweden | 4 | 4 | 1 | 2 | 3 | 3 | 3 | 4 | 3 | 27 |
| Kirkham EJ *et al*, 2022, UK | 4 | 4 | 4 | 4 | 4 | 4 | 4 | 4 | 4 | 36 |
| Grande D *et al*, 2022, USA | 4 | 4 | 4 | 3 | 4 | 4 | 3 | 4 | 3 | 33 |
| Bosanac D *et al*, 2022, Croatia | 2 | 4 | 2 | 1 | 3 | 2 | 3 | 4 | 2 | 23 |
| Nunes Vilaza G *et al*, 2021, Denmark | 4 | 4 | 3 | 3 | 4 | 3 | 4 | 4 | 3 | 32 |
| Beesley SJ *et al*, 2022, USA | 2 | 3 | 3 | 3 | 4 | 3 | 3 | 4 | 2 | 27 |
| Braunack-Mayer A *et al*, 2021, Australia | 3 | 4 | 3 | 4 | 4 | 3 | 4 | 4 | 4 | 33 |
| Nong P *et al*, 2021, USA | 3 | 2 | 4 | 2 | 3 | 2 | 3 | 4 | 2 | 25 |
| Helou S *et al*, 2021, USA, Japan | 4 | 4 | 2 | 1 | 4 | 1 | 3 | 4 | 2 | 25 |
| Brall C *et al*, 2021, Switzerland | 2 | 3 | 3 | 4 | 4 | 4 | 3 | 4 | 3 | 30 |
| Tosoni S *et al*, 2021, Canada | 4 | 2 | 2 | 1 | 2 | 2 | 3 | 4 | 2 | 22 |
| Yu E *et al,* 2021, USA | 4 | 4 | 4 | 4 | 4 | 3 | 3 | 4 | 3 | 33 |
| Vidgen ME *et al*, 2020, Australia | 4 | 4 | 2 | 2 | 3 | 2 | 3 | 4 | 4 | 28 |
| Middleton A *et al*, 2020, Argentina | 2 | 4 | 2 | 4 | 3 | 4 | 3 | 4 | 4 | 30 |
| Weis A *et al*, 2020, Germany | 3 | 4 | 3 | 2 | 4 | 2 | 3 | 4 | 4 | 29 |
| Woldaregay AZ *et al*, 2020, Norway, Swiss, USA | 2 | 3 | 3 | 3 | 3 | 4 | 4 | 4 | 2 | 28 |
| Esmaeilzadeh P *et al*, 2020, USA | 3 | 3 | 3 | 4 | 4 | 3 | 2 | 4 | 1 | 27 |
| Udesky JO *et al*, 2020, USA | 2 | 3 | 3 | 3 | 4 | 3 | 3 | 4 | 3 | 28 |
| Hah H. *et al*, 2020, USA | 3 | 4 | 4 | 2 | 3 | 3 | 4 | 4 | 4 | 31 |
| Middleton A *et al*, 2020, USA, UK | 2 | 2 | 2 | 4 | 4 | 4 | 2 | 4 | 3 | 27 |
| Karampela M *et al* 2019, Europe | 3 | 3 | 4 | 4 | 4 | 4 | 4 | 4 | 2 | 32 |
| Soni H *et al*, 2020, USA | 4 | 4 | 4 | 2 | 3 | 2 | 3 | 4 | 4 | 30 |
| Jackman KP *et al*, 2021, USA | 4 | 4 | 4 | 2 | 4 | 3 | 4 | 4 | 4 | 33 |
| Milne R *et al,* 2019, UK, USA | 3 | 3 | 3 | 4 | 4 | 3 | 3 | 4 | 2 | 29 |
| Soni H *et al*, 2019, USA | 2 | 4 | 3 | 2 | 4 | 2 | 3 | 4 | 3 | 27 |
| Kim J *et al*, 2019, USA | 4 | 3 | 3 | 2 | 3 | 2 | 3 | 4 | 4 | 28 |
| Smith TG *et al*, 2019, USA | 4 | 4 | 2 | 3 | 3 | 4 | 4 | 4 | 3 | 31 |
| Seltzer E *et al*, 2019, USA | 3 | 4 | 2 | 2 | 3 | 2 | 3 | 4 | 3 | 26 |
| Mahmoud R *et al*, 2019, USA | 4 | 3 | 3 | 3 | 4 | 3 | 3 | 4 | 3 | 30 |
| Esmaeilzadeh P *et al*, 2019 | 3 | 4 | 4 | 3 | 4 | 3 | 4 | 4 | 4 | 33 |
| Krahe M *et al*, 2019, Australia | 4 | 3 | 3 | 3 | 4 | 3 | 4 | 4 | 4 | 32 |
| Kim TK *et al*, 2019, Australia | 4 | 4 | 2 | 2 | 4 | 2 | 2 | 4 | 2 | 26 |
| Weng C *et al*, 2019, USA | 3 | 4 | 2 | 2 | 3 | 2 | 3 | 4 | 3 | 26 |
| Esmaeilzadeh P *et al*, 2018, USA | 3 | 4 | 4 | 3 | 4 | 3 | 3 | 4 | 3 | 31 |
| Vaala SE *et al*, 2018, USA | 4 | 4 | 3 | 2 | 3 | 3 | 4 | 4 | 4 | 31 |
| Kim KK *et al*, 2017, USA | 4 | 4 | 1 | 2 | 4 | 2 | 3 | 4 | 2 | 26 |
| Sanderson SC *et al,* 2017, USA | 2 | 3 | 3 | 4 | 4 | 3 | 3 | 4 | 3 | 29 |
| Cheung C *et al,* 2016, USA | 2 | 3 | 2 | 2 | 3 | 2 | 3 | 4 | 2 | 23 |
| Medford-Davis LN *et al,* | 3 | 4 | 2 | 2 | 3 | 2 | 3 | 4 | 3 | 26 |
| Serrano KJ *et al,* 2016, USA | 3 | 4 | 3 | 3 | 4 | 3 | 3 | 4 | 3 | 30 |
| Yaraghi N *et al,* 2015, USA | 1 | 3 | 1 | 3 | 1 | 1 | 2 | 4 | 1 | 17 |
| Mataya L *et al,* 2015, USA | 3 | 3 | 2 | 3 | 4 | 3 |  | 4 | 2 | 24 |
| Grande D *et al,* 2015, USA | 3 | 3 | 4 | 3 | 4 | 3 | 4 | 4 | 3 | 31 |
| Kim KK *et al,* 2015, USA | 3 | 3 | 3 | 3 | 4 | 3 | 3 | 4 | 3 | 29 |
| Frost J *et al,* 2014, Netherland | 3 | 2 | 3 | 2 | 3 | 1 | 4 | 3 | 4 | 25 |
| Kitayama K *et al,* 2014, USA | 4 | 3 | 2 | 1 | 1 | 3 | 2 | 2 | 2 | 20 |
| Grande D *et al,* 2013, USA | 4 | 3 | 4 | 2 | 3 | 3 | 4 | 3 | 2 | 28 |
| Weitzman ER *et al,*2012, USA | 4 | 2 | 4 | 4 | 4 | 3 | 4 | 3 | 1 | 29 |
| Maiorana A *et al,* 2012, USA | 4 | 4 | 3 | 3 | 4 | 1 | 3 | 2 | 3 | 27 |
| Patel VN *et al,* 2011, USA | 4 | 4 | 4 | 3 | 3 | 1 | 3 | 3 | 3 | 28 |
| Teixeira PA *et al,* 2011, USA | 4 | 2 | 2 | 2 | 4 | 1 | 3 | 1 | 2 | 21 |
| Haddow G *et al,* 2011, UK | 4 | 4 | 2 | 1 | 2 | 1 | 2 | 1 | 2 | 19 |
| Weitzman ER *et al,* 2010, USA | 4 | 4 | 2 | 1 | 2 | 1 | 3 | 1 | 2 | 20 |
| Hunter IM *et al,* 2009, New Zeland | 2 | 2 | 3 | 4 | 3 | 1 | 4 | 4 | 2 | 25 |
| Simon SR *et al,* 2009, USA | 4 | 2 | 3 | 1 | 2 | 1 | 3 | 2 | 2 | 20 |
| Whiddett R *et al,* 2006, New Zeland | 1 | 3 | 2 | 2 | 4 | 2 | 4 | 2 | 4 | 24 |
| Sun S. *et al,* 2022, China | 2 | 4 | 4 | 3 | 4 | 1 | 3 | 2 | 2 | 25 |
| Garett R *et al,* 2022, USA | 3 | 2 | 2 | 1 | 1 | 3 | 3 | 1 | 1 | 17 |
| Halmdienst N *et al,* 2022, Austria | 2 | 4 | 4 | 4 | 4 | 1 | 3 | 2 | 2 | 26 |
| Matthews E *et al,* 2022, USA | 4 | 4 | 4 | 1 | 2 | 1 | 4 | 3 | 2 | 25 |
| Holm S *et al,* 2021, Denmark | 2 | 4 | 3 | 2 | 4 | 2 | 4 | 1 | 1 | 23 |
| Belfrage S *et al,* 2021, Sweden | 2 | 4 | 3 | 2 | 2 | 4 | 4 | 2 | 2 | 25 |
| Hentschel A *et al,* 2021, USA | 4 | 2 | 3 | 2 | 2 | 2 | 3 | 1 | 3 | 22 |
| Lu C *et al,* 2020, Canada | 4 | 2 | 4 | 1 | 3 | 1 | 4 | 1 | 2 | 22 |
| Bussone A *et al,* 2020, UK | 2 | 4 | 3 | 1 | 1 | 1 | 2 | 1 | 4 | 19 |
| Wagner L *et al,* 2020, USA | 3 | 2 | 1 | 2 | 2 | 2 | 2 | 1 | 1 | 16 |
| Esmaeilzadeh P*,* 2020, USA | 3 | 3 | 3 | 1 | 4 | 3 | 4 | 1 | 3 | 25 |
| Bouras A *et al,* 2020, USA | 4 | 4 | 2 | 1 | 3 | 1 | 4 | 2 | 2 | 23 |
| Khan F.A. *et al* 2020, Saudi Arabia*,* | 2 | 2 | 2 | 1 | 2 | 1 | 3 | 1 | 2 | 16 |
| Juga J *et al,* 2020, Finland | 4 | 3 | 2 | 1 | 2 | 1 | 2 | 1 | 2 | 18 |
| Navarro-Millán I *et al,* 2019, USA | 4 | 2 | 4 | 4 | 4 | 1 | 2 | 2 | 2 | 25 |
| Calero Valdez A *et al,* 2019, Germany | 2 | 3 | 2 | 1 | 3 | 2 | 4 | 2 | 2 | 21 |
| Atchariyachanvanich K *et al,* 2018, Thailand | 3 | 3 | 3 | 1 | 3 | 1 | 3 | 1 | 3 | 21 |
| Goodman D *et al,* 2017, USA | 3 | 1 | 3 | 2 | 3 | 3 | 3 | 4 | 2 | 24 |
| Lucero R.J. *et al,* 2015, USA | 3 | 3 | 3 | 3 | 4 | 1 | 2 | 3 | 1 | 23 |
| Li T. *et al* 2014 Netherland*,* | 3 | 2 | 2 | 1 | 3 | 1 | 2 | 2 | 2 | 18 |
| Kimura M. *et al,* 2014, Japan | 4 | 3 | 2 | 1 | 2 | 1 | 4 | 1 | 3 | 21 |
| Grant A *et al,* 2013, UK | 4 | 4 | 4 | 1 | 3 | 2 | 2 | 1 | 4 | 25 |
| Anderson C.L. *et al,* 2011, USA | 2 | 4 | 3 | 3 | 2 | 2 | 3 | 1 | 4 | 24 |
| Green B.B *et al,* 2011, USA | 4 | 2 | 4 | 1 | 2 | 1 | 4 | 2 | 2 | 22 |
| Bansal G. *et al,* 2010, USA | 2 | 4 | 4 | 1 | 3 | 2 | 4 | 1 | 4 | 25 |
| Cherif E. *et al,* 2021, France | 4 | 3 | 3 | 2 | 3 | 1 | 3 | 1 | 3 | 23 |
| Al-Khalifa M. *et al,* 2016, Saudi Arabia | 2 | 3 | 4 | 1 | 2 | 1 | 2 | 1 | 3 | 19 |
| Wu B.G. *et al,* 2022, China | 2 | 3 | 2 | 1 | 1 | 1 | 1 | 1 | 1 | 13 |
| Trinidad M.G. *et al,* 2020, USA | 2 | 4 | 2 | 1 | 3 | 1 | 4 | 1 | 4 | 22 |
| Lysaght T. *et al,* 2020, Singapore | 4 | 3 | 3 | 1 | 2 | 1 | 2 | 2 | 1 | 19 |

**Supplementary Table 1.** Quality assessment.

## Supplementary Table 2

| **Author, Year, Country** | **Aim** | **Study designs** | **Population, sample** | **Secondary users** | **Main findings** | **Recruitment** | **Recruiter** | **Context** | **Statistical analysis** |
| --- | --- | --- | --- | --- | --- | --- | --- | --- | --- |
| Lee CMY *et al.,* 2022, Australia | To describe the willingness of consumers to use digital technology for health and to share their health information. | survey | Community sample, 1778 | Research, commercial | Digital awareness and education are crucial to virtual medicine implementation. | N.A. | Market research companies | online | inferential statistics |
| Tosoni S *et al,* 2022, Canada | To describe differences in consent preferences between pre-pandemic times and the first wave of Covid-19. | interview | Oncologic patients, 183 | Research, commercial | During the pandemic, patients were more comfortable sharing information with all parties, except with commercial entities, where levels of discomfort remained unchanged. | N.A. | Researchers | online | inferential statistics, qualitative content analysis |
| Cherif E *et al,* 2022, France | To investigate patients’ perceived benefits and privacy concerns related to EHRs to develop different user profiles and propose practical measures for public policymakers. | survey | Community sample, 1076 | Public-policy makers | Privacy concerns and perceived risk were found to significantly hinder the willingness to adopt HER. Trust, perceived usefulness, and perceived control were significant motives for adoption. | social networks / mailing lists / posted fliers | Researchers | online | cluster analysis |
| Belfrage S *et al*, 2022, Sweden | To examine how the general public’s trust relates to their attitudes toward the use of health data. | survey | Community sample, 1645 | EHR | Trust is crucial for the broad use of health data for a variety of socially valuable purposes. | N.A. | Public / no-profit organizations | online | descriptive statistics |
| Kirkham EJ *et al*, 2022, UK | To investigate factors influencing the likelihood of sharing these data for research purposes amongst people with and without experience with mental illness. | survey | People with experience of mental illness, 2187 | Research | Higher satisfaction with the national health systems is associated with a greater willingness to share mental health data. People with experience of mental illness are more willing than people without mental illness to share their mental health data once satisfaction is considered. | social networks / mailing lists / posted fliers | Researchers | online | inferential statistics |
| Grande D *et al*, 2022, USA | To find out factors associated with consumers’ willingness to share their digital information for health-related uses. | survey | Community sample, 3542 | EHR | Although consumers’ willingness to share personal digital information for health purposes is associated with the context of use, many have underlying privacy views that affect their willingness to share. | N.A. | Market research companies | online | conjoint analysis, generalized estimating equation model |
| Bosanac D *et al*, 2022, Croatia | To find out how much Croatian trust e-Health systems and their willingness to share personal health data. | survey | Community sample, 102 | EHR, research | The use of the e-Health Portal in Croatia remains low, although there is a high level of trust in confidentiality, anonymity, and willingness to share data for research. | N.A. | Market research companies | online | inferential statistics |
| Nunes Vilaza G *et al*, 2021, Denmark | To examine public attitudes to research repositories storing samples and personal health data and identifying implications for future developments. | survey | Community sample of Brazilian and Denmark young adults, 1600 | Research | The key factors for acceptance of personal health data sharing include transparency about the goals and beneficiaries of research projects, control over data access, and awareness of the benefits of data sharing for research. | social networks / mailing lists / posted fliers | Researchers | online | inferential statistics |
| Beesley SJ *et al*, 2022, USA | To understand the information-sharing preferences of former intensive care unit patients and their family members and to identify predictors of information-sharing preferences. | survey | Patients and their relatives, 1470 | EHR, research | In the context of an intensive care unit admission, sharing personal health information with a person of the patient's choosing appears desirable for most patients and family members. | N.A. | Market research companies | online | inferential statistics |
| Braunack-Mayer A *et al*, 2021, Australia | To explore public attitudes in Australia toward sharing government health data with the private sector. | interview | Community sample of adolescents and adults, 2537 | Research, commercial | Australians are uncertain about sharing their health data with the private sector. There is strong support for strict conditions on sharing data and for opt-in consent and significant concerns about how well the private sector would manage health data. | N.A. | Market research companies | online | inferential statistics, qualitative content analysis |
| Nong P *et al*, 2021, USA | To analyze trust in public health sources of information and willingness to participate in public health efforts by race, specifically on Black-White racial differences. | interview | Community sample of ethnic groups, 1000 | EHR | Black survey respondents reported a higher willingness to participate in public health efforts related to COVID-19 than White respondents in the unadjusted analysis. In the adjusted analysis, there was no statistically significant difference between Black and White respondents’ willingness to participate. | N.A. | Public / no-profit organizations | online | explanatory sequential mixed method |
| Helou S *et al*, 2021, USA, Japan | To analyze how the perceived usefulness, sensitivity, and anonymity of personal health data relate to people's willingness to share it with researchers. | survey | Community sample, 112 | Research | People are more willing to share their health data with researchers if they perceive it as useful for public health research, not sensitive, and trust that they cannot be identified after sharing it. | social networks / mailing lists / posted fliers | Researchers | online | inferential statistics |
| Brall C *et al*, 2021, Switzerland | To identify motives, concerns, and expectations of the Swiss public about providing health information and biological material for personalized health research. | survey | Community sample, 5136 | Research, commercial | Personalized health research is supported by slightly more than half of the Swiss public, but exist concerns about discrimination, confidentiality, and misuse of data for commercial purposes | N.A. | Public / no-profit organizations | online, in-person session | inferential statistics |
| Tosoni S *et al*, 2021, Canada | To acquire an in-depth understanding of the contemporary and specific consent needs of cancer patients at a large academic hospital to inform its institutional consent policies. | survey | Oncologic patients, 222 | Research, commercial | Patients want to decide the conditions under which PHI may be shared, whether to track their secondary use, and whether to receive results of studies using PHI. | attending people | Researchers | in-person sessions | inferential statistics |
| Yu E *et al,* 2021, USA | To explore the willingness to share medical information among patients with multiple sclerosis (MS), who experience higher rates of psychiatric comorbidities compared to the general population, and the role that stigma plays in patient preferences. | survey | Members of Multiple Sclerosis Association of America, 3020 | EHR | Participants were slightly more willing to share their non-psychiatric medical information compared to psychiatric information. Patients with psychiatric co-occurring disorders, compared to those without, endorsed significantly greater willingness to electronically share their health records. Societal stigma strongly correlated with decreased non-psychiatric medication sharing, while self-stigma was strongly correlated with decreased psychiatric medication sharing. | N.A. | Public / no-profit organizations | online | inferential statistics |
| Vidgen ME *et al*, 2020, Australia | To explore public opinions related to the sharing of genomic data for research from clinical records in the context of current policies. | survey | Community sample, 1494 | Research, commercial | Some health information management policies do not fulfill the expectation of participants for genomics data sharing. | social networks / mailing lists / posted fliers | Researchers | online | inferential statistics, qualitative content analysis |
| Middleton A *et al*, 2020, Argentina | To explore global public attitudes toward the willingness to donate one’s DNA and health information to be shared for research (both non-profit and for-profit), together with an understanding of the factors that shape this. | survey | Community sample, 36268 | Research, commercial | The results demonstrated the importance of familiarity with the implication of genomic research and trust in the collection and sharing of genomic and health data. | N.A. | Market research companies | online, in-person session | random effects model |
| Weis A *et al*, 2020, Germany | To explore mHealth usage, preferences, barriers, and eHealth literacy reported by German patients with rheumatic diseases. | survey | Rheumatologic patients, 193 | EHR, research | A substantial majority of patients are willing to share app data for research purposes. | N.A. | Public / no-profit organizations | in-person sessions | qualitative content analysis |
| Woldaregay AZ *et al*, 2020, Norway, Swiss, USA | To examine factors related to people's knowledge and expectations toward raising motivation for sharing health-related data. | survey | People with and without diabetes, 447 | Research | Concern about health data sharing is dependent on the type of data. | N.A. | Researchers | online | descriptive statistics |
| Esmaeilzadeh P *et al*, 2020, USA | To examine the influence of structured and unstructured data entry format on perceptions of individuals suffering from mental and physical illnesses on PHI sharing. | interview, hypothetical scenarios | Patients, 607 | EHR, research | Respondents with chronic physical diseases were more willing to share information when structured interfaces were presented to them. | N.A. | Market research companies | online | inferential statistics |
| Udesky JO *et al*, 2020, USA | To assess (a) how the data-sharing method affects willingness to participate in environmental studies, (b) how prospective research participants perceive the risks and benefits of such studies, and (c) whether views vary with contextual factors, namely the study purpose, type of data, and approach to the return of study results. | survey, hypothetical scenarios | Breast cancer patients, 1575 | EHR, Research | Participants prefer controlled access to children’s data. They are more reluctant to share locations or to participate if studies involve electronic medical records. Many express concerns about privacy. | social networks / mailing lists / posted fliers | Researchers | online | inferential statistics, qualitative content analysis |
| Hah H. *et al*, 2020, USA | To examine health consumers’ existing habits regarding financial information management of the willingness to share health data in various scenarios. | survey | Community sample, 339 | EHR, Research | Frequent users of financial technology are more prone to share their entire health information in all instances, even with non–care-related stakeholders. | N.A. | Market research companies | online | propensity score matching, average treatment effect |
| Middleton A *et al*, 2020, USA, UK | To examine whether “genetic exceptionalism”, defined as the belief that DNA information is different from other forms of medical information, is associated with the willingness or unwillingness to donate data. | survey | People with exceptionalistic view on genetic data, 8967 | Research | Offering to return results in some form, providing clear information about legal protections, and engaging that addresses the distinctive characteristics of genetic information may encourage data sharing. Specific risks from re-identification alone did not appear to dissuade the public from being willing to participate in research. | N.A. | Market research companies | online | inferential statistics |
| Karampela M *et al* 2019, Europe | To explore user attitudes toward sharing personal health data. The study was executed within the first year after the implementation of the new General Data Protection Regulation legal framework. | survey | Community sample, 8004 | Research | Most users are willing to share their health data for scientific research. Age, education level, and occupation of the participants, in addition to the level of digitalization in their country, were found to be associated with data-sharing attitudes. | N.A. | Market research companies | online | descriptive statistics |
| Soni H *et al*, 2020, USA | To assess content sensitivity and preferences for granular data sharing for care and research. | interview, hypotetical scenarios | Patients, 61 | EHR, research | Participants considered mental health, sexual and reproductive health and alcohol use, and alcoholism-sensitive information. Participants were willing to share information related to other addictions, genetic data, and general physical health information. | attending people | Researchers | in-person sessions | descriptive statistics |
| Jackman KP *et al*, 2021, USA | To describe, using mixed methods, perceptions of access to sexually transmitted infection test results via electronic personal health record (PHR) and correlates of willingness to adopt its use. | survey, focus groups | 18-25 years old black students, 354 | EHR, research | Data highlights interest among Black college-age youth in adopting personal health records for comprehensive sexual health-related services. | social networks / mailing lists / posted fliers | Researchers | in-person sessions | explanatory sequential mixed method |
| Milne R *et al,* 2019, UK, USA | To explore trust in genomic data sharing among members of the public in the UK; USA; Canada and Australia. | survey | Community sample, 8967 | EHR, research, commercial | Trust, acknowledging, and being transparent about different users of genomic data reinforce the willingness to share health data. | N.A. | Market research companies | online | latent class analysis, inferential statistics |
| Soni H *et al*, 2019, USA | To survey English and Spanish-speaking behavioral health patients, including those with SMIs, on their perceptions regarding data sensitivity, willingness to share health data for care and research, and related motivations. | interview | People suffering from behavioral conditions, 86 | EHR, research, commercial | The attitudes towards data sharing depend on the perspectives on data sharing and privacy. | attending people | Researchers | in-person sessions | inferential statistics |
| Kim J *et al*, 2019, USA | To assess patient perspectives about decisions to share medical data and biospecimens for research. | survey | Patients, 1246 | EHR | A tiered-permission system that allowed for the specific removal of data items or categories of data mattered to participants with whom the health data and biospecimens would be shared. Data and biospecimen sharing preferences were different according to the opting method. | attending people | Researchers | in-person sessions | inferential statistics |
| Smith TG *et al*, 2019, USA | To examines cancer survivors' perspectives about sharing patient health data with central cancer registries. | interview, focus groups | Cancer patients and survivors, 52 | EHR, research | Registry-based collection of patients' health data is acceptable to most cancer survivors and could facilitate registry-based efforts to collect data. Central cancer registry-based collection of health data could enhance registry support of cancer control efforts including research and population health management. | N.A. | Public / no-profit organizations | via-telephone, in-person session | grounded theory approach |
| Seltzer E *et al*, 2019, USA | To credibly evaluate participants’ willingness to share data, the health relatedness of those digital data sources, and preferences associated with data sharing. | survey | Patients, 206 | EHR, research | Participants would be willing to donate some of their digital data to researchers and clinicians in pursuit of health-related insights. | attending people | Researchers | in-person sessions | exploratory factor analysis |
| Mahmoud R *et al*, 2019, USA | To understand patients' attitudes to sharing their imaging data for research purposes. | survey | Patients, 798 | Research | People are willing to donate their medical images for research studies after full consent that establishes privacy, security, and control over permission and duration of access. Respondents are more willing to donate their data to universities and research institutions. | attending people | Researchers | in-person sessions | inferential statistics |
| Esmaeilzadeh P *et al*, 2019 | To explore the core value of blockchain technology in the healthcare industry from patients’ views. | survey, hypothetical scenarios | Community sample, 1128 | EHR | Blockchain technology has great potential to be integrated into existing health information exchange architectures to improve system transparency, patient consent tracking, and privacy protection of information exchange initiatives. | N.A. | Market research companies | online | inferential statistics |
| Krahe M *et al*, 2019, Australia | To explore the opinions, perceived risks, and trustworthiness regarding the use of personal health information for research, in a sample of the public attending a tertiary healthcare facility. | survey | Community sample, 249 | Research | Building trust among the community on system and technology credibility, establishing confidence in the research, and addressing concerns related to sensitivity and privacy may increase social sustainability in health data sharing. | attending people | Researchers | in-person sessions | inferential statistics, qualitative content analysis |
| Kim TK *et al*, 2019, Australia | To explore factors influencing older adults’ willingness to share their personal and health information when using healthcare technologies and services. | survey | Older adults, 170 | EHR, research, commercial | Most older adults reported being willing to share their information with family and hospitals than with researchers, government agencies, device developers, or insurance companies. Higher education consistently showed a negative effect on the willingness to share, and older age showed a positive effect. Older adults with positive attitudes towards technology were more likely to share their information with device developers/corporations. Women were less likely to share their information with government agencies than men. Older adults with poorer self-rated health were more likely to share their information with hospitals. | attending people | Researchers | in-person sessions | inferential statistics |
| Weng C *et al*, 2019, USA | To investigate the degree to which well-informed individuals might be willing to share clinical information and to characterize the degree to which individuals would share specific clinical content. | survey | Students and staff at major medical centers, 1764 | EHR, research | A substantial fraction of potential patient participants once educated about risks and benefits, would be willing to donate de-identified clinical data to a shared research repository. A slight majority even would be willing to share absent de-identification, suggesting that perceptions about data misuse are not a major concern. | social networks / mailing lists / posted fliers | Researchers | online | inferential statistics |
| Esmaeilzadeh P *et al*, 2018, USA | To investigate how patients’ preferences regarding information exchange (i.e., privacy concern, opt-in intention, and perceived health information sensitivity) are affected by different HIE models and exchange architectures. | survey, hypothetical scenarios | Community sample, 1416 | EHR | There are significant differences in patients’ perceptions of different HIE mechanisms in terms of privacy concerns and opt-in intention. Consumers believe that the patient-controlled HIE is the most preferred model to protect health. | N.A. | Market research companies | online | inferential statistics |
| Vaala SE *et al*, 2018, USA | To examine factors underlying adolescents’ willingness to share personal health information with peers. | survey | Adolescents suffering from type 1 diabetes, 134 | EHR | Adolescents were more willing to share how they accomplished diabetes tasks than how often they completed them, and least willing to share glucose control status. Sharing/helping beliefs and glucose control were related to a greater willingness to share personal health information. Adolescents with worse glucose control had stronger relationships between sharing/helping beliefs and willingness to share but weaker relationships between helping experience and willingness to share. | attending people | Researchers | online | inferential statistics |
| Kim KK *et al*, 2017, USA | To explore factors that affect consumers’ willingness to share electronic health information for healthcare and research. | survey, focus groups | Community sample, 100 | EHR, research | Consumers’ choices about electronically sharing health information are affected by their attitudes toward EHRs as well as beliefs about research benefits and individual control. | via telephone | Market research companies | via-telephone, in-person session | inferential statistics |
| Sanderson SC *et al,* 2017, USA | To assess willingness to participate in a biobank using different consent and data sharing models, hypothesizing that willingness would be higher under more restrictive scenarios. | survey, hypothetical scenarios | Patients, 13000 | Research | Some socio-demographic groups differ in their willingness to participate in biobank research. Targeted interventions designed to recruit underrepresented groups, to make biobank information easier to understand, and to address individuals’ specific attitudes about participating in a biobank may help increase acceptance of broad consent and open data sharing in biobank research. | attending people | Researchers | via-letter, online | inferential statistics |
| Cheung C *et al,* 2016, USA | To understand the privacy attitudes of early adopters of emerging health technologies. | interview | Early technology adopters, 11 | EHR, research | Although interviewees were willing to share personal data to support scientific advancements, they still expressed concerns, as well as uncertainty about who has access to their data, and for what purpose. | N.A. | Public / no-profit organizations | in-person sessions | grounded theory approach |
| Medford-Davis LN *et al,* | To determine whether emergency department patients want to share their medical records across health systems through Health Information Exchange and if so, whether they prefer to sign a consent or share their records automatically. | interview | Patients of an emergency department, 982 | EHR | Study results show that most patients are in favor of HIE, and that consent to access HIE records should be explored for inclusion in the informed consent forms. | attending people | Researchers | in-person sessions | inferential statistics |
| Serrano KJ *et al,* 2016, USA | To examine willingness to exchange different types of health information via mobile devices and assessed whether sociodemographic characteristics and trust in clinicians were associated with willingness in a nationally representative sample. | survey | Community sample, 3165 | EHR | Respondents were less willing to exchange via mobile devices information that may be considered sensitive or complex. Age, socioeconomic factors, and trust in professional information were associated with willingness to engage in mobile health information exchange. | N.A. | Researchers | online | inferential statistics |
| Yaraghi N *et al,* 2015, USA | To empirically explore the drivers of patients' consent to share their medical records on health information exchange platforms. | informed consent and patients' choice analysis | Patients, 18055 | EHR | The likelihood of providing consent to sharing health data increases with age. Female patients are more likely to provide consent. As the number of different physicians involved in the care of the patient increases, the odds of providing consent slightly increases. The odds of providing consent are significantly higher for the patients for whom a primary care physician has been involved in their medical care | attending people | Researchers | N.A. | inferential statistics |
| Mataya L *et al,* 2015, USA | To explore the attitudes of kidney donors and recipients regarding how much information they believe should be shared. | survey, hypothetical scenarios | Kidney donors and recipients, 392 | HIE | Both kidney donors and recipients want a significant amount of health information to be disclosed. | N.A. | Public / no-profit organizations | online | inferential statistics |
| Grande D *et al,* 2015, USA | To understand patient views on the reuse of health information. | survey | People with and without history of cancer, 2945 | EHR, research, commercial | The information-sharing preferences of participants with and without a prior diagnosis of cancer were driven mainly by the purpose of information reuse. Participants with cancer were more willing to share their inherited genetic information. | N.A. | Market research companies | online | main effect analysis of variance |
| Kim KK *et al,* 2015, USA | To analyze consumers' views on privacy, security, and consent in electronic data sharing for healthcare and research together. | survey | Community sample, 800 | EHR, research | People’s views on sharing data for research are affected by privacy, security, trust in the organization conducting the research, seeing the information, purpose, and kind of research. Participants were more willing to share data for research than healthcare. | via telephone | Market research companies | via-telephone | inferential statistics |
| Frost J *et al,* 2014, Netherland | To document patient preferences for sharing information within online health platforms. | interview | Cancer patients, 115 | HIE | Respondents’ information-sharing intentions with peers depend on dispositional and situational factors. Patients share medical details more willingly than daily life or identity information. The results suggest the need to focus on anonymity rather than privacy in online communities. | social networks / mailing lists / posted fliers | Researchers | online | principal component analysis |
| Kitayama K *et al,* 2014, USA | To examine desired characteristics of an online immunization record for parents from a predominantly Latino, low-income population. | survey, focus groups | Low-income community sample, 29 | EHR | If privacy concerns are adequately addressed, parents of low-income, urban children are likely to use and benefit from an online immunization record. | attending people | Researchers | in-person sessions | qualitative content analysis |
| Grande D *et al,* 2013, USA | To measure patient preferences about sharing their electronic health information for secondary purposes. | survey, hypothetical scenarios | Community sample, 3336 | EHR, research, commercial | Participants cared most about the specific purpose for using their health information, although differences were smaller among racial and ethnic minorities. The user of the information was of secondary importance, and the sensitivity was not a significant factor. | N.A. | Market research companies | online | main effect analysis of variance |
| Weitzman ER *et al,*2012, USA | To investigate parent and young adult attitudes toward sharing personal health information with clinical and public health decision-makers. | survey | Pediatric patient's parents, 261 | EHR, research | Pediatric patients and their families are often willing to share electronic health information to support health improvement. | attending people | Researchers | online | inferential statistics |
| Maiorana A *et al,* 2012, USA | To examine trust in technological systems, operational procedures, and people influence the acceptability of data sharing among patients, providers, and other stakeholders. | interview | People living with HIV, 549 | HIE | Patients and providers are willing to accept the electronic sharing of HIV patient data to improve. Acceptability depends on the effort expended to understand and address potential concerns related to data sharing and confidentiality, and on the trust established among stakeholders. | attending people | Researchers | in-person sessions | inferential statistics, qualitative content analysis |
| Patel VN *et al,* 2011, USA | To examine consumers’ support for physicians’ use of HIE and their potential usage of EHRs in a low-income, ethnically diverse community. | survey | Low-income patients and caregivers, 214 | HIE | Most low-income ethnically diverse consumers support HIE. | attending people | Researchers | in-person sessions | inferential statistics |
| Teixeira PA *et al,* 2011, USA | To assess the attitudes of persons living with HIV/AIDS towards having their personal health information stored and shared electronically. | survey | Insurance client living with HIV, 93 | EHR | Most people living with HIV were willing to share personal health data. Trust and communications with providers are important factors. | attending people | Researchers | in-person sessions | inferential statistics |
| Haddow G *et al,* 2011, UK | To explore lay views about the anonymization and data-sharing process. | interview, focus groups | Community sample, 19 | Research | There were specific concerns about control and access and a generalized skepticism and mistrust of the government and large commercial and insurance organizations. | N.A. | Researchers | in-person sessions | inductive approach |
| Weitzman ER *et al,* 2010, USA | To characterize consumer willingness to share personally collected health records data for health research and the conditions and contexts bearing on willingness to share. | interview | Community sample, 151 | Research | Strong support for sharing personal health information for health research existed among early adopters of the EHR. | N.A. | Researchers | online | inferential statistics, qualitative content analysis |
| Hunter IM *et al,* 2009, New Zeland | To investigate public attitudes towards sharing personal health information. | survey | Community sample, 1828 | Research | The level of identification of personal health data, who is requesting info, purpose, and how much info is sought are the determinants. | via telephone | Researchers | via-telephone | inferential statistics, cluster analysis |
| Simon SR *et al,* 2009, USA | To explore patient view about info sharing and their preferences | survey, focus groups | Patients, 64 | EHR | Patients are enthusiastic about electronic health information exchange; however, they are also concerned about its potential to result in breached privacy and misuse of health data. | social networks / mailing lists / posted fliers | Researchers | in-person sessions | qualitative content analysis |
| Whiddett R *et al,* 2006, New Zeland | To investigates the attitudes of patients toward sharing info | survey | Adult primary-care patients, 189 | EHR, research, commercial | Many respondents were unwilling to have their personal information distributed other than for purposes of clinical care. | attending people | Researchers | in-person sessions | inferential statistics |
| Sun S. *et al,* 2022, China | To explore the relationship amongst the factors, the security control of the platform, perceived usefulness, information sensitivity, privacy concerns in platforms, patients' satisfaction, trust, and disclosure intention. | survey | Community sample, 500 | EHR, research, commercial | Trust in a sharing platform does not positively influences patients’ disclosure intention, while satisfaction with online healthcare communities does. | N.A. | Researchers | in-person sessions | structural equation model |
| Garett R *et al,* 2022, USA | To examine participants' social media use and comfort in sharing their data with health researchers. | survey | Patients, 161 | EHR | More than one-third of participants reported being very comfortable sharing electronic health data and social media data for personalized healthcare and to help others | social networks / mailing lists / posted fliers | Researchers | online | inferential statistics |
| Halmdienst N *et al,* 2022, Austria | To examine the personal health situation and how the complexities thereof affect the elderly Austrians’ willingness to accept electronic health records. | survey | Elder people, 3103 | EHR | The higher the degree of multimorbidity, the more medication is prescribed, and the higher the number of hospital admissions, the higher the acceptance of EHR. Having a chronic illness has a positive effect on EHR acceptance, whereas a pessimistic attitude and lack of joy in life, as indicators of depressive mood, have a negative impact. | N.A. | Market research companies | N.A. | generalized estimating equation model |
| Matthews E *et al,* 2022, USA | To identify patient barriers and facilitators towards the acceptance of HIE within the context of depression treatment and to examine how HIE impacts depression-related care coordination and patient activation. | interview | Patients suffering depression, 27 | HIE | While HIE was perceived to improve the overall quality of depression care, the stigma associated with mental illness undermined the more robust adoption of this technology among underserved populations. | attending people | Researchers | via-telephone, via-video | qualitative content analysis |
| Holm S *et al,* 2021, Denmark | To study patients' trust, their interest in being asked about secondary use, and their willingness to dispense from a requirement to informed consent based on their trust. | survey | Community sample, 994 | Research | Even in a population that has a high level of trust in the handling of health data for research, there is still a significant proportion of citizens who want some form of control over how their data are used and exchanged. | N.A. | Public / no-profit organizations | online | inferential statistics, Jonckheere-Terpstra test for trend |
| Belfrage S *et al,* 2021, Sweden | To investigate what views are held by the public, and what aspects matter for the willingness to let one’s data be used not only for one’s care but also for other purposes | survey | Community sample, 1629 | EHR, HIE, research, commercial | The great majority of the respondents have a positive attitude towards the use of their electronic health data, both for purposes relating to their health and for purposes primarily benefitting others, but also that they want to be able to influence the use of their health data and have it protected from unauthorized access. | N.A. | Researchers | online | descriptive statistics |
| Hentschel A *et al,* 2021, USA | To identify factors affecting their willingness to participate in research | interview | Pregnant or breastfeeding woman,29 | EHR, research | Participants were largely familiar with the engagement of their EHR for health care purposes, and most of them were willing to release their EHRs to researchers, provided their concerns for privacy, confidentiality, and transparency were addressed | social networks / mailing lists / posted fliers | Researchers | in-person sessions | exploratory factor analysis |
| Lu C *et al,* 2020, Canada | To explore the intentions and concerns of healthcare consumers regarding the adoption of blockchain-based personal health records and data sharing | interview, focus groups | Community sample, 26 | EHR, EHI, research, commercial | The study did not show strong intentions among healthcare consumers to adopt blockchain-based health data sharing. | social networks / mailing lists / posted fliers | Researchers | in-person sessions | qualitative content analysis |
| Bussone A *et al,* 2020, UK | To assess how people living with HIV conceptualize trust, privacy, and security for sharing data with others | interview | People living with HIV, 26 | HIE | The development of trustworthy and secure digital platforms that enable people living with HIV to share data with their peers facilitates data sharing. | social networks / mailing lists / posted fliers | Public / no-profit organizations | in-person sessions | inferential statistics, qualitative content analysis |
| Wagner L *et al,* 2020, USA | To explore preferences for sharing EHRs for research among young adults suffering from autism spectrum disorder, fragile X syndrome, or neurodevelopmental disorders | interview, focus groups | Adults with autism spectrum disorder or fragile X syndrome, 44 | EHR, research | This study has highlighted conditional support for EHR research among those with autism spectrum disorders or fragile X syndrome. While young adults with neurodevelopmental disorders were generally willing to participate in EHR research | N.A. | Public / no-profit organizations | in-person sessions | qualitative content analysis, kappa coefficient of agreement |
| Esmaeilzadeh P*,* 2020, USA | To analyzes the effects of cognitive trust and emotional trust on the intention to opt-in to HIEs and willingness to disclose health information | survey | Community sample, 493 | HIE | Both cognitive and emotional procedures can determine the extent to which patients will rely on the HIE | N.A. | Market research companies | online | structural equation model |
| Bouras A *et al,* 2020, USA | To assess healthy non-Hispanic white mothers' attitudes in five areas: motivation to share data, concern with data use, desire to keep health information anonymous, use of patient portal, and willingness to share anonymous data with researchers. | survey | Non-Hispanic white women, 622 | EHR, research, commercial | Mothers’ motivation to contribute to science is the principal driver of data-sharing intentions. Mothers’ access to the internet and the utilization of patient portals did not have a significant effect on their willingness to disclose their health data. | N.A. | Market research companies | online | inferential statistics |
| Khan F.A. *et al* 2020, Saudi Arabia*,* | To analyze the awareness and willingness of Saudi Arabians the use EHR. | survey | Community sample, 164 | EHR | Age, family history, and type of disease are significant factors in willingness to adopt the EHR. While computer skills, compatibility of the EHR system, and access to the EHR system are insignificant associations with the adoption of EHR | N.A. | Researchers | N.A. | inferential statistics |
| Juga J *et al,* 2020, Finland | To explicate the behavioral factors that determine willingness to share personal health data for secondary uses | survey | Community sample aged 15-79, 2338 | EHR, HIE, research, commercial | Attitude is the strongest behavioral determinant of the willingness to share PHI for secondary uses. However, also trust and control play an important mediating role in mitigating the privacy concerns related to the sharing of sensitive information such as personal health data. | N.A. | Market research companies | online | principal factor axis method, maximum likelihood method |
| Navarro-Millán I *et al,* 2019, USA | To identify the perspectives of patients with rheumatoid arthritis on the electronic recording of between-visit disease activity and other patient-reported outcomes and on sharing this information with healthcare providers or peers. | interview, focus groups | Rheumatoid Arthritis patients, 31 | EHR, HIE | Patients with rheumatoid arthritis may be amenable to electronic collection and sharing of data between clinical encounters if it facilitates communication with health care providers. | attending people | Researchers | in-person sessions | qualitative content analysis |
| Calero Valdez A *et al,* 2019, Germany | To investigate the importance and utility of privacy-preserving techniques related to sharing of personal health data. | survey, focus groups | Community sample, 539 | EHR, HIE, research, commercial | Users disagreed with sharing data for commercial purposes regarding mental illnesses and with high de-anonymization risks but showed little concern when data is used for scientific purposes and is related to physical illnesses | social networks / mailing lists / posted fliers | Researchers | in-person sessions | cojoint analysis |
| Atchariyachanvanich K *et al,* 2018, Thailand | To explore the factors that affect personal health information disclosure via a mobile application in Thailand. | survey | Community sample, 294 | Commercial | Privacy concern was not significantly negatively related to the intention to disclose personal health information. However, the significant effect of the perceived benefit, personalized service, and self-presentation was consistent with previous studies. | social networks / mailing lists / posted fliers | Researchers | online | structural equation model |
| Goodman D *et al,* 2017, USA | To measure the preferences regarding the addition of data to a research repository and views about the oversight and sharing of de-identified genomic data in a group of research. | survey | People with and without cancer, 904 | Research | The majority of participants were not concerned about personal identification when participating in a genetic study using de-identified data. Most participants expressed a desire that their data should be available for as many research studies as possible. | via letter | Researchers | online | inferential statistics |
| Lucero R.J. *et al,* 2015, USA | To explore community members’ views on the secondary use of digitized clinical data to recruit participants for research. | survey, focus groups | Community sample, 30 | Research | The overriding concern of community members regarding secondary clinical and nonclinical use of digitized information was that their involvement would be safe, and the outcome would be meaningful to them and to others. | attending people | Public / no-profit organizations | in-person sessions | qualitative content analysis |
| Li T. *et al* 2014 Netherland*,* | To provide new insights into how privacy concerns play a role in patients’ decisions to permit the digitization of their personal health information. | survey | People with familiarity with Electronic Health Records, 160 | EHR | The results suggest that giving patients the greater ability to control their information can alleviate their privacy concerns when they make opt-in decisions. | N.A. | Researchers | online | structural equation model |
| Kimura M. *et al,* 2014, Japan | To clarify the views of the general population of two countries (US and Japan), concerning the handling of their medical records electronically. | survey | Community sample, 200 | EHR, research, commercial | US participants sustained that the extent of sharing their identifiable medical records should be limited to the doctors in charge and specified doctors referred to by their doctors. Japanese people find it acceptable for doctors of the same hospital to share their medical records. | N.A. | Market research companies | N.A. | inferential statistics |
| Grant A *et al,* 2013, UK | To explore whether a register of volunteers interested in research participation, with data linkage to electronic health records to identify suitable research participants, would prove acceptable to healthcare staff, patients, and researchers. | interview, focus groups | Practitioners, their patients, and research staff, 64 | EHR, HIE, research | Patients, health service staff, and researchers have a favorable view of the potential benefits of a national register held within the public sector to identify people who are potentially eligible and willing to participate in health-related research. | via telephone | Researchers | in-person sessions | qualitative content analysis |
| Anderson C.L. *et al,* 2011, USA | To assess under what circumstances individuals are willing to disclose identified personal health information and permit it to be digitized. | interview, hypothetical scenarios | Community sample, 1000 | EHR, HIE, research, commercial | The contextual factors related to the requesting stakeholder and the purpose for which the information has been requested play an important role in moderating privacy concerns. Emotions play a pivotal role in health data disclosure. | N.A. | Market research companies | N.A. | inferential statistics |
| Green B.B *et al,* 2011, USA | To know whether subgroups, particularly those from vulnerable populations, are less willing to participate or unable to because they lacked computer access. | survey | Patients suffering hypertension, 7354 | Research | Older age, lower socioeconomic status, and lower levels of education were associated with decreased access to and willingness. | via telephone | Researchers | via-telephone | inferential statistics |
| Bansal G. *et al,* 2010, USA | To understand the role of personal dispositions in disclosing health information online. | survey | Students, 367 | EHR | Personal dispositions should be taken into consideration when examining privacy concerns and behavioral intentions to disclose health information online | social networks / mailing lists / posted fliers | Researchers | online | exploratory factor analysis, structural equation model |
| Cherif E. *et al,* 2021, France | To introduce personal health concerns and trust in healthcare providers as new predictors. | survey | People with various degrees of e-health literacy, 584 | EHR, research | Health concerns and trust in healthcare providers have a great role in determining the intention to create an EHR and to share personal health data | social networks / mailing lists / posted fliers | Researchers | online | structural equation model |
| Al-Khalifa M. *et al,* 2016, Saudi Arabia | To identify different factors that determine patients’ acceptance of sharing their medical information among different care providers. | survey | Saudi and non-Saudi residences, 300 | EHR | Perceived benefits and perceived risks are the two factors that influence the attitude of patients to share their health information. | social networks / mailing lists / posted fliers | Researchers | online | structural equation model |
| Wu B.G. *et al,* 2022, China | To explore the impact of users’ health information privacy concerns on their engagement in online health communities and the moderating effects of users’ benefit and threat appraisal for information-sharing behaviors | survey | University students, 480 | HIE | There is a negative relationship between users’ health information privacy concerns and their online health community engagement. | N.A. | Market research companies | online | structural equation model |
| Trinidad M.G. *et al,* 2020, USA | To assess comfort with sharing health data with third-party commercial companies for patients. | survey | Community sample, 1841 | Commercial | Educational attainment is associated with increased comfort with sharing health data with third-party commercial companies for patient purposes and decreased comfort with sharing health data with third-party commercial companies for business purposes, and privacy concern is strongly associated with less comfort with sharing health data with third-party commercial companies for both patient and business purposes. | N.A. | Public / no-profit organizations | online | inferential statistics |
| Lysaght T. *et al,* 2020, Singapore | To examine the ethical concerns Singaporeans have about sharing health data for precision medicine. | interview, focus groups | Community sample, 62 | EHR, Research | Maintenance of public trust in data security systems and governance regimes can enhance participation in data sharing for research. Participants demonstrated a sophisticated understanding of the inherent risks of data sharing. | N.A. | Market research companies | online | qualitative content analysis |

**Supplementary Table 2.** Studies description and main results.
